# Safe From Harm: Learned, Instructed, and Symbolic Generalization Pathways of Human Threat-Avoidance

**DOI:** 10.1371/journal.pone.0047539

**Published:** 2012-10-15

**Authors:** Simon Dymond, Michael W. Schlund, Bryan Roche, Jan De Houwer, Gary P. Freegard

**Affiliations:** 1 Department of Psychology, Swansea University, Swansea, United Kingdom; 2 Department of Behavioral Psychology, Kennedy Krieger Institute, Baltimore, Maryland, United States of America; 3 Department of Psychiatry, Johns Hopkins University School of Medicine, Baltimore, Maryland, United States of America; 4 Department of Behavior Analysis, University of North Texas, Denton, Texas, United States of America; 5 Department of Psychology, National University of Ireland, Maynooth, Co. Kildare, Ireland; 6 Department of Psychology, Ghent University, Ghent, Belgium; University of New South Wales, Australia

## Abstract

Avoidance of threatening or unpleasant events is usually an adaptive behavioural strategy. Sometimes, however, avoidance can become chronic and lead to impaired daily functioning. Excessive threat-avoidance is a central diagnostic feature of anxiety disorders, yet little is known about whether avoidance acquired in the absence of a direct history of conditioning with a fearful event differs from directly learned avoidance. In the present study, we tested whether avoidance acquired indirectly via verbal instructions and symbolic generalization result in similar levels of avoidance behaviour and threat-beliefs to avoidance acquired after direct learning. Following fear conditioning in which one conditioned stimulus was paired with shock (CS+) and another was not (CS−), participants either learned or were instructed to make a response that cancelled impending shock. Three groups were then tested with a learned CS+ and CS− (learned group), instructed CS+ (instructed group), and generalized CS+ (derived group) presentations. Results showed similar levels of avoidance behaviour and threat-belief ratings about the likelihood of shock across each of the three pathways despite the different mechanisms by which they were acquired. Findings have implications for understanding the aetiology of clinical avoidance in anxiety.

## Introduction

Avoidance of potentially threatening or unpleasant events is an often-automatic feature of daily life. Sometimes, avoidance has obvious survival advantages, for instance, in situations where someone heeds a warning call and avoids the path of an oncoming car. However, there may be countless other occasions where avoidance is the default mechanism for coping with fearful events, such as when a socially anxious individual avoids new social situations because of the potentially aversive consequences that may occur. The predominant use of avoidance as a means of attenuating the effects of actual and anticipated fear can lead to further social withdrawal. Moreover, by definition, engaging in avoidance allows for few, if any, opportunities to disconfirm the hypothesis that avoidance may actually be counterproductive. As a result, avoidance can become chronic and debilitating and lead to impaired social functioning and possible psychopathology.

Excessive avoidance is a central diagnostic feature and known risk factor in the acquisition and maintenance of anxiety disorders [1]–[4]. Clinical and anecdotal reports of anxiety often, but not always, describe a prior experience of fear conditioning that precipitates avoidance [5], [6]. Fear conditioning involves pairing a conditioned stimulus (CS; e.g., a light) with an unconditioned stimulus (US; e.g., electric shock). After sufficient pairings, presentations of the CS alone come to elicit conditioned fear [7]. While it widely known that stronger fear conditioning may be a factor in anxiety disorders [8], a growing body of evidence also attests to the fact that direct contact with an aversive event may not be necessary for fear conditioning to occur [9]–[12], [6]. Indeed, fear acquired through pathways other than Pavlovian conditioning, such as verbal instructions and social observation, is often indistinguishable from directly learned fear that arises following pairing of neutral and aversive stimuli [13]–[16]. Further analysis of fear and avoidance acquired via indirect pathways may provide a novel basis for understanding the aetiology of anxiety disorders [5], [17].

Regardless of how it is acquired, fear often leads to expectancies and avoidance of fear provoking or threatening stimuli. Avoidance occurs when a response, such as pressing a key, in the context of a warning signal (e.g., a light) that precedes a US leads to omission of the US. By virtue of Pavlovian conditioning, the warning signal comes to function as a CS and elicits fear because of its prior relationship with the US when the avoidance response is not made. Avoidance is learned via operant negative reinforcement when the probability of emitting the avoidance response in the context of the warning signal increases [18]. While it is likely that some avoidance behaviours are acquired following direct contact with an aversive stimulus, many forms of chronic avoidance may be acquired through indirect pathways [19]. Despite this, surprisingly little research has been conducted on these alternative pathways and whether they result in equivalent levels of avoidance. Thus, an investigation into the relative levels of avoidance in the presence of CSs acquired via indirect pathways, such as instruction, and following direct aversive Pavlovian conditioning would be salutary.

Within associative learning, stimulus generalization may occur whereby stimuli physically similar to the CS occasion responding along a specified dimension. For instance, fear conditioning has been shown to generalize along perceptual [9], [20], [21] and conceptual/semantic continua [10], [22]. Little is known, however, about the generalization of avoidance along symbolic dimensions. Whereas generalized avoidance of degraded coloured circles along a physical continuum between CS+ and CS− has been demonstrated [23], other evidence suggests that *symbolic generalization* of avoidance may also occur in which physically dissimilar stimuli indirectly related to the CS occasion avoidance [24]–[30].

Symbolic generalization is underpinned by an extensive literature on derived stimulus relations, such as stimulus equivalence, which shows that when humans are taught a series of relations involving dissimilar (arbitrary) stimuli, the stimuli involved often become related to each other in ways not explicitly trained [31], [32]. For instance, if choosing Stimulus X in the presence of Stimulus A is taught (i.e., A–X), and choosing Stimulus Y in the presence of Stimulus A (i.e., A–Y) is also taught, it is likely that untrained relations will emerge between X and A, Y and A, X and Y, and Y and X, in the absence of any feedback. Moreover, when one stimulus is also a CS for avoidance the remaining, indirectly related stimuli may also come to occasion symbolic generalization of avoidance in the absence of any further learning [18]. For instance, Dymond and colleagues [27] first trained and tested participants for the formation of stimulus equivalence relations consisting entirely of abstract, arbitrary stimuli (AV1-AV2-AV3-AV4 and N1-N2-N3-N4; note that AV refers to cues from the class of avoidance stimuli, and N refers to neutral cues). During the avoidance-learning phase, one stimulus (AV2) was followed by aversive images and sounds unless a response was made (pressing the space-bar once), and another (N2) was not. When the avoidance response was performed, AV2 was removed from the screen and the aversive stimuli omitted. The symbolic generalization of this threat-avoidance responding was then tested with presentations of stimuli that had not been present during the avoidance-learning phase. All participants readily made the threat-avoidance response to AV3 and AV4 (indirectly related to AV2) and not to N3 and N4 (indirectly related to N2). Additionally, measures of threat beliefs (subject’s expectancies of aversive images following avoidance and non-avoidance) paralleled avoidance behavior patterns, thereby providing the first evidence of symbolic generalization of threat beliefs.

The findings of Dymond et al. showed that establishing avoidance of one stimulus in a derived relational network also produces avoidance of all indirectly related stimuli in the network, via symbolic generalization. It remains to be seen, however, whether avoidance rates and expectancies of an aversive stimulus (i.e., threat beliefs) acquired via symbolic generalization are comparable to those acquired via other pathways, such as instructions, and to what extent they differ, if at all, from directly learned avoidance.

According to propositional models of avoidance learning [33], [34], avoidance behaviour is driven by propositional beliefs about when aversive events are likely to occur and how those aversive events can be avoided. Importantly, propositions can be formed not only on the basis of direct experience but also on the basis of instructions or inferences. As such, propositional models allow for avoidance behaviour that is based on instructions (via propositions that result from instructions) and symbolic generalization (via propositions that are formed as the result of inferences). To the extent that instructions and inferences contain the same information as direct experience, all three routes should give rise to identical propositions and would thus lead to similar threat-belief ratings and avoidance responses. Note that from the perspective of propositional models, symbolic generalization in avoidance learning requires more inferential steps than the other pathways. Participants not only need to form the proposition that a particular stimulus (e.g., AV2) is followed by a US, but also that another stimulus is equivalent to that CS (e.g., AV4) and will thus also be followed by a US. Assuming that an error or uncertainty might occur at each inferential step, symbolic generalization in avoidance learning could turn out to be weaker that avoidance learning via direct observation or instructions.

To test this, the present experiment employed a between-participants design to investigate the acquisition and maintenance of avoidance through *learned, instructed*, and *derived* pathways. We hypothesized that the groups would not differ following fear conditioning in that threat-belief ratings of the likelihood of shock following a CS+ would always be greater relative to a CS-. After avoidance learning, we expected all groups to make a greater proportion of avoidance responses to a CS+ relative to a CS−, give lower threat-belief ratings in the presence of the avoidance response, and higher ratings in the absence of the avoidance response, to the CS+ relative to the CS−. We predicted that this trend would be maintained during extinction testing and that levels of avoidance and threat beliefs occasioned by the Instructed CS+ and Derived CS+ would not differ.

## Materials and Methods

### Participants

Ninety participants, 22 men and 68 women (*M* age = 25.06, *SD* = 10.05) were recruited from Swansea University and randomly assigned to one of three groups: Learned, Instructed, and Derived. All participants provided written informed consent and were compensated with either course credit or £5.

### Ethics Statement

The Department of Psychology, Ethics Committee, Swansea University approved the study. All participants provided signed, informed consent.

### Apparatus and Material

The experiment was conducted in a small room containing a desktop PC and a 17″ monitor with a 60 Hz refresh rate. Coloured circles (red, blue, yellow, and green) served as CSs; two were used for the Learned group, three for the Instructed group, and four for the Derived group, respectively. Electric shocks delivered via a bar electrode fitted to the participant’s non-dominant forearm were controlled by a PowerLab® isolated stimulator (ADI Instruments, Oxford, United Kingdom). At the outset, all groups underwent a shock calibration procedure in which they selected a shock level that was “uncomfortable, but not painful”.

### Procedure

**Figure 1:**
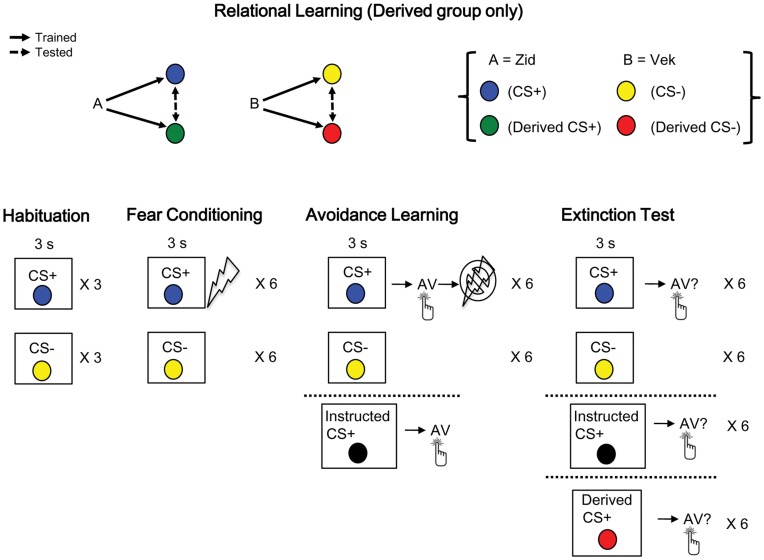
Schematic overview of experimental design. All groups received habituation, fear conditioning, avoidance learning, and extinction test phases. The Derived group only received the relational learning phase prior to habituation. Colours were counterbalanced across participants. Hatched lines in avoidance learning and extinction test phases indicate additional stimuli presented to either the Instructed or Derived group. See text for details.

All groups received the same four conditioning phases (habituation, fear conditioning, avoidance learning, and extinction test; see Fig. 1). Prior to the fear and avoidance phases, only participants in the Derived group were exposed to a relational learning phase involving nonsense words (A = “Zid”, B = “Vek”) and coloured circles (counterbalanced across participants) as stimuli. For this group, a matching-to-sample procedure was used to train conditional discriminations (Zid-Blue, Zid-Green, Vek-Yellow, Vek-Red) and test for the emergence of stimulus equivalence relations (Blue-Green, Green-Blue, Yellow-Red, Red-Yellow; see Fig. 1). On every training trial, a nonsense word (Zid or Vek) first appeared in the top centre of the computer screen (called the sample stimulus). Clicking on the sample immediately produced two coloured circles (Blue and Yellow or Green and Red) positioned below and to the left and right of the screen (called the comparison stimuli). Participants selected one of the comparisons by clicking on it with the computer mouse. When Zid was presented, clicking on the Blue comparison stimulus produced the feedback “Correct” in the centre of the screen, while clicking on Yellow produced the feedback “Wrong”. When Vek was presented, clicking on the Yellow comparison stimulus produced the feedback “Correct” in the centre of the screen, while clicking on Blue produced the feedback “Wrong”. When Zid was presented, clicking on the Green comparison stimulus produced the feedback, “Correct” in the centre of the screen, while clicking on Red produced the feedback “Wrong”. When Vek was presented, clicking on the Red comparison stimulus produced the feedback, “Correct” in the centre of the screen, while clicking on Green produced the feedback “Wrong” (see Fig. 1). Feedback was displayed in size 14 Arial black font within a 4.5×2 cm square in the middle of the screen for 2 s, and was followed by an intertrial interval (ITI) of 2 s. All four tasks (Zid-Blue, Zid-Green, Vek-Yellow, Vek-Red) were presented in a block of 8 trials (each task presented twice) in a pseudorandom order, with the constraint that the same task could not appear on more than two consecutive trials. Correct selections were taught via feedback until participants made eight consecutive correct responses.

On meeting the training criterion, a block of 16 trials were presented that tested for the emergence of combined symmetry and transitivity (i.e., stimulus equivalence) relations. Each of the four tasks (Blue-Green, Green-Blue, Yellow-Red, Red-Yellow) was presented four times in the absence of feedback. When Blue was presented, clicking on the Green comparison not Red, when Vek was presented, clicking on the Yellow comparison not Blue, when Zid was presented, clicking on the Blue comparison not Yellow, and when Vek was presented, clicking on the comparison Red not Green, was predicted (Fig. 1). The mastery criterion was 16 consecutive correct responses; if necessary, participants were re-exposed to training and testing until the criterion was met. For the Derived group, relational training and testing created stimulus relations consisting of coloured circles to be used in the subsequent phases.

In the *habituation* phase, participants were told that on each trial one of two coloured circles would appear and that they should simply watch the screen. The CS+ and CS− were each presented 3 times for 3 s in the absence of shock. Order of presentation was quasi-randomized with the constraint that no more than two consecutive trials of either type could occur. In the *fear conditioning* phase, participants were informed that on every trial they would be presented with one of two coloured circles and that each circle would appear for 3 s followed by either a 250 ms shock or no shock. Participants were told the shock was set at the level they had selected and that they would be asked to make some ratings about the likelihood of shock following each of the coloured circles. Each CS appeared 6 times in the centre of the screen for 3 s and shock was presented following offset of the CS+. Shock never followed any CS− presentations. A 6 s ITI always occurred. After the 12^th^ trial, participants rated the likelihood of shock following the CS+ and CS− using a 6-point scale (where 0 = *not at all* and 6 = *very likely*).

Next, in the *avoidance learning* phase, the Learned and Derived groups were informed that when coloured circles appeared on screen the marked keys on the keyboard would be available and that pressing one of the keys (participants were not told which) in the presence of one coloured circle would cancel upcoming shock. They were also told that the key that cancelled shocks was the same on all trials (this was counterbalanced across participants). The Instructed group was also informed that when the Instructed CS+, which was a coloured circle not presented during fear conditioning, appeared they should press the specified marked key on the right to avoid upcoming shock. For the Learned and Derived groups, blocks of 12 trials, 6 of each CS, were presented and participants had to meet the criterion of a minimum of 5 out of 6 CS+ trials with the avoidance response to complete this phase. Only one key could be pressed on each trial; when the correct key was pressed, the CS+ was removed from the screen and the ITI initiated, and when the incorrect key was pressed shock always followed CS+ but not CS− trials. Pressing the correct key had no scheduled effects in the presence of the CS− (i.e., it remained on screen). No criterion was applied to the CS−, and the 12-trial block was repeated until criterion was met.

For the Instructed group, blocks of 18 trials were presented (i.e., CS+, CS− and Instructed CS+ each presented 6 times in a quasi-random order) and participants had to meet the criterion of a minimum of 5 out of 6 CS+ trials with the avoidance response within a 18-trial block, which was repeated until criterion was met. Although no formal criterion was applied to the Instructed CS+, when the correct key was pressed in the presence of the Instructed CS+, the stimulus was removed from the screen and the ITI was initiated. Pressing the incorrect key in the presence of the Instructed CS+ was, however, followed by shock. Because of the possibility that participants may directly experience shock after the Instructed CS+ when pressing the incorrect key, only those participants who made the avoidance response on all Instructed CS+ presentations were included in the analysis. After the final trial, threat-belief ratings were made of the likelihood of shock following the CS+, CS− and, where relevant, Instructed CS+ and Derived CS+, when the avoidance response was and was not assumed to have been performed.

The final phase was the *extinction test* (i.e., no shocks were presented), which began immediately following avoidance learning. For all groups, the CS+ and CS− were each presented 6 times. The Instructed group also received 6 presentations of the Instructed CS+, while the Derived group received 6 trials of the Derived CS+, respectively (Fig. 1). Once again, participants rated the likelihood of shock following the CS+ and CS−, and, where relevant, the Instructed CS+ and Derived CS+, in the assumed presence and absence of avoidance, respectively.

### Data Analysis

During fear conditioning, mean ratings of the likelihood of shock following the CS+ and CS− were measured, while during the avoidance learning and extinction test phases the total mean number of trials in which the avoidance response was and was not performed, the number of cycles of trial blocks taken to meet criterion, and mean ratings of the likelihood of shock following the CS+, CS−, Instructed CS+ and Derived CS+, with and without the avoidance response, were recorded. For the Derived group only, additional dependent measures were the mean number of trials to reach training criterion and the mean number of stimulus equivalence test exposures. Multivariate *F* values, Pillai’s trace, are reported for all main effects and interactions, with stimulus and pathway as factors. Analyses were performed for avoidance behavior and ratings with and without avoidance. Finally, paired sample *t*-tests were conducted within groups to assess differences between the Direct CS+ and each pathway. For all tests, level of significance was set at.05.

## Results

Participants in the Derived group required a mean of 73.5 (*SD* = 63.7) training trials to reach criterion and a mean of 1.63 (*SD* = 1.1) exposures to pass the equivalence test.

Analysis of threat belief ratings provided the index of fear conditioning, and revealed a significant main effect of stimulus (*F*_(2,87)_ = 3149.02, *p*<.001), but no interaction between stimulus and group (*p* = .692). As predicted, post-hoc *t*-tests showed that CS+ and CS− ratings differed significantly in the Learned, *t*_(29)_ = 20.796, *p*<.001, Instructed, *t*_(29)_ = 13.88, *p*<.001, and Derived, *t*_(29)_ = 24.39, *p*<.001, groups, respectively. This shows that each of the three pathways groups had formed a clear threat-belief of shock following the CS+ but not following the CS−.

One participant in the Learned group and 3 from the Instructed group failed to meet criterion in the avoidance learning phase and were removed from further analysis. The final sample sizes were: Direct (*n = *29), Instructed (*n = *27), and Derived (*n = *30).

**Figure 2:**
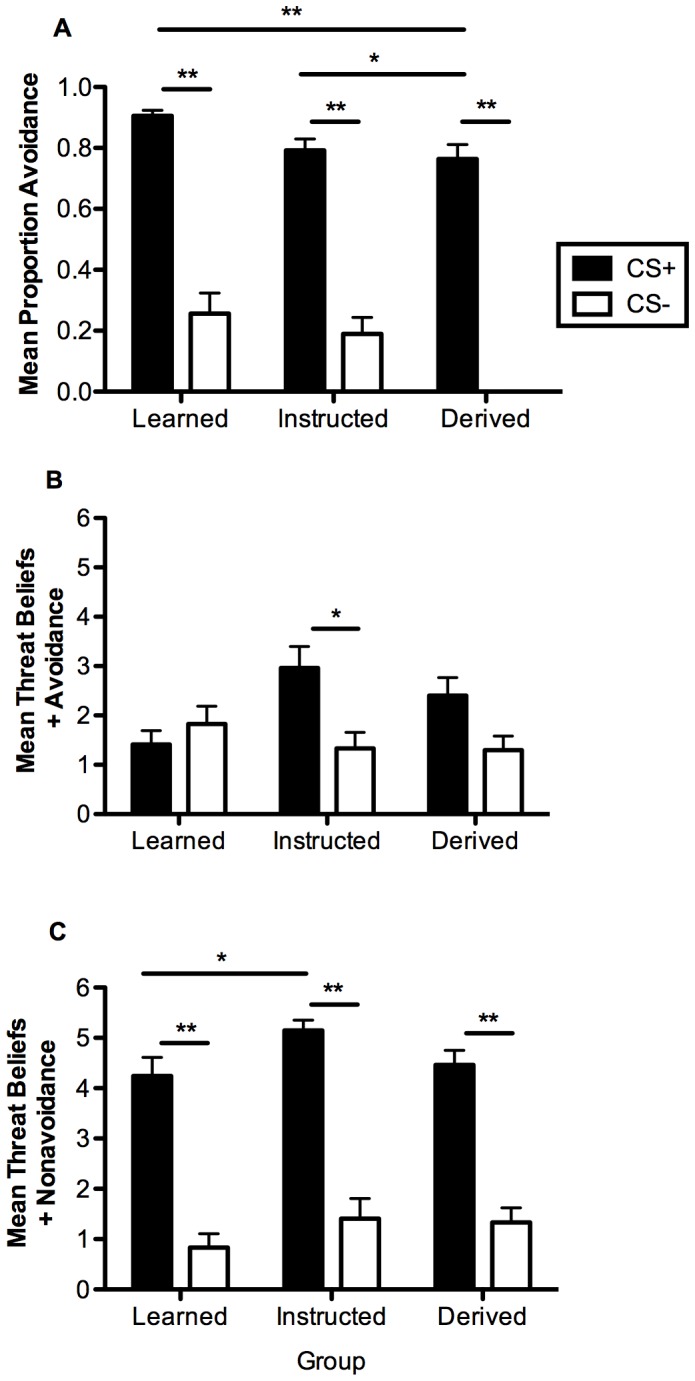
Avoidance learning results. (A) Mean proportion of avoidance in each of the groups. (B) Mean threat-beliefs with avoidance. (C) Mean threat-beliefs without avoidance. Error bars reflect standard error of the mean (SEM). * *P*<0.05, ** *P*<0.001.

During avoidance learning (Fig. 2), we found a significant main effect of stimulus [*F*(1,83) = 379.151, *p*<.001], which is consistent with our hypotheses regarding a greater proportion of avoidance responding to a threatening CS+ compared to a CS−, and no interaction (*p* = .078). Subsequent paired sample *t*-tests confirmed that there was a greater proportion of avoidance of the CS+ than the CS− by each group (all *p*’s <0.001). The absence of a significant interaction with group illustrates that avoidance performed in the presence of the CS+ was comparable regardless of the pathway. Subsequent post-hoc tests (corrected) revealed that the proportion of avoidance responses evoked by the CS+ differed between the Learned and Derived (*p*<.001) and Instructed and Derived groups (*p*<.05).

In the analysis of threat-beliefs, we found a significant main effect of stimulus when ratings questions asked participants to assume they had [*F*_(1,83)_ = 5.72, *p*<.05] or had not performed the avoidance response [*F*_(1,83)_ = 123.036, *p*<.001]. These findings indicate that during avoidance learning all groups similarly expected the presence or absence of shock depending on which CS was present. There was a significant interaction between group and stimulus when avoidance was assumed to be present [*F*_(1,83)_ = 3.590, *p*<.05] but not when absent (*p = *.713). Follow-up tests showed no differences in in threat belief ratings made when the avoidance response was assumed to be present (all *p’*s >.05). With ratings made in the absence of avoidance, only the Learned and Instructed groups differed in (*p*<.05).

**Figure 3:**
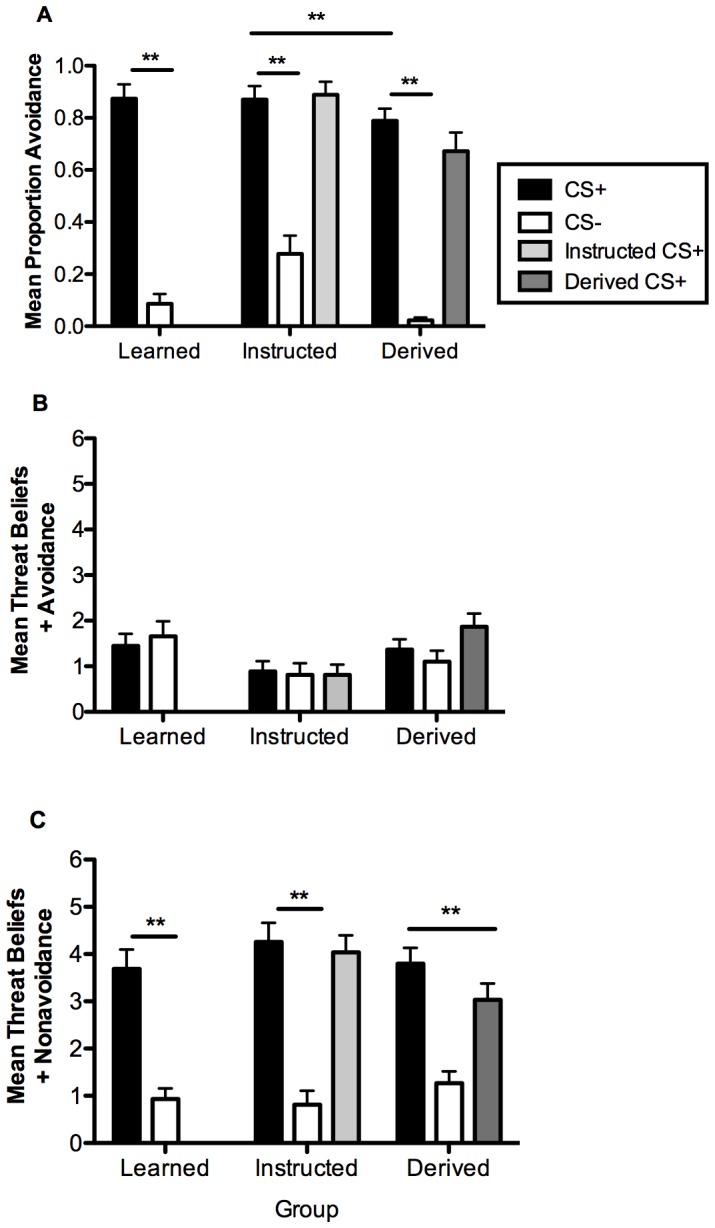
Extinction test results. (A) Mean proportion of avoidance. (B) Mean threat-beliefs with avoidance. (C) Mean threat-beliefs without avoidance. Error bars reflect standard error of the mean (SEM). * *P*<0.05, ** *P*<0.001.

Analysis of the proportion of avoidance responses evoked by the CS+ and CS− during the extinction test phase (Fig. 3) showed there was a main effect of stimulus, *F*_(1,83)_ = 307.729, *p*<.001, indicating greater avoidance of the CS+ than CS−, but no interaction with group (*p = *.115). Subsequent paired sample *t*-tests confirmed a greater proportion of avoidance of the CS+ than the CS− by each group (all *p*’s <.001). Again, these findings are consistent with our hypotheses concerning the maintenance of avoidance acquired via learned, instructed and derived pathways when tested under extinction. Follow-up analyses revealed that only the Instructed and Derived groups differed (*p*<.001) in the proportion of avoidance evoked by the CS+. Moreover, there were no differences in avoidance evoked by the directly learned CS+ and the Instructed CS+ (*p = *.302) and the directly learned CS+ and the Derived CS+ (*p* = .158).

When threat-belief ratings were made when the avoidance response was assumed to be present, we found no main effect of stimulus (*p = *.810) and no interaction (*p* = .570). Figure 3 shows that ratings were uniformly low in each of the three groups for all stimuli presented. When ratings were made in the assumed absence of avoidance, then a main effect [*F*_(1,83)_ = 104.993, *p*<.001] was found with higher ratings given to the CS+ than CS−, but no significant interaction (*p* = .407). These findings mirror those from avoidance learning (Fig. 2) and extend them to situations involving ratings of the instructed and derived CSs. No significant differences were found in follow-up analyses of threat beliefs made when the avoidance response was assumed to be either present or absent (all *p’*s >0.05). Finally, threat beliefs made to the directly learned CS+ and the Instructed CS+ when the avoidance response was assumed to be present (*p* = .713) and absent (*p = *.456) did not differ, while threat beliefs made to the directly learned CS+ and the Derived CS+ also did not differ when avoidance was present (*p* = .158), but did when it was assumed to be absent, *t*_(30)_ = 2.715, *p*<.05.

## Discussion

Our findings demonstrate, for the first time, the acquisition and maintenance of equivalent levels of avoidance behaviour acquired via learned, instructed and symbolic generalization (derived) pathways. In line with our predictions, avoidance behaviour of the three groups did not differ following fear conditioning and avoidance learning, and threat-belief ratings of the likelihood of shock following the CS+ were always greater relative to the CS−. Consistent with our predictions, these trends persisted during extinction testing as all groups made a greater proportion of avoidance responses to the CS+ relative to the CS− and gave lower threat-belief ratings in the presence of the avoidance response and higher ratings in the absence of the avoidance response to the CS+ compared to the CS−. Moreover, levels of avoidance and threat beliefs occasioned by the Instructed CS+ and Derived CS+ did not differ. Taken together, these findings show that conditioned, instructed and derived pathways to threat-avoidance and threat-beliefs in humans are equally efficacious [14].

During avoidance learning, avoidance evoked by the directly learned CS+, which all participants experienced from the outset, differed between the Learned and Derived and Instructed and Derived groups, respectively, with the latter difference also evident in the subsequent test phase. The factors responsible must for the present time remain speculative, but it is notable that the Instructed CS+ occasioned more avoidance than the Derived CS+, demonstrating the powerful effect that verbal instructions exert over behaviour. By comparison, the relatively similar but non-significantly different level of avoidance occasioned by the Derived CS+ is consistent with our previous findings on human symbolic generalization of avoidance [25]–[27]. It was also notable that avoidance occasioned by the Learned CS+ and the Derived CS+ was the only significant difference observed across the groups during testing (Fig. 3), with avoidance of the directly learned CS+ in each of the instructed and derived groups being broadly similar. The reduction in levels of avoidance occasioned by the Derived CS+ has been widely observed [27] and is partly due to the fact that testing is undertaken under conditions of extinction. While participants avoided approximately 70% of all Derived CS+ presentations and presumably treated it as if it were equivalent to a learned CS+, it is possible that having not encountered it since the relational learning phase its presentation could have prompted participants to initially withhold avoidance responding in order to determine whether or not shocks would be delivered. However, this possibility is unlikely because a re-examination of the raw data revealed that only 30% of participants in the Derived group failed to make the correct avoidance response from the very first presentation of the Derived CS+. This suggests that for the majority of participants in this group, presentation of a cue indirectly related to a learned CS+ for avoidance was sufficient to evoke generalized avoidance from the outset. Similarly, the differences found between the directly learned CS+ and Derived CS+ (Fig. 3) indicate that for the Derived group, threat-beliefs were likely modulated by the combined influence of the novel testing context and extinction conditions. It remains to be seen whether extended extinction testing would serve to diminish avoidance responding and beliefs still further. Future research on the persistence of derived avoidance and test factors influencing relative rates of instructed avoidance is clearly warranted.

During testing, the proportion of avoidance evoked by the CS+ only differed between the Instructed and Derived groups (a trend that continued from acquisition), but, crucially, the Instructed CS+ and Derived CS+ did not differ from the Learned CS+. That is, avoidance evoked by a cue that was directly learned about, instructed, or indirectly related to a CS+ was indistinguishable and occurred under conditions where no aversive events (shocks) were scheduled. The present findings are the first demonstration of equivalent levels of avoidance occasioned by each of three different pathways and add to the existing literature on social transmission of fear [14], the role of verbal instructions in human learning [35], [36] and processes of symbolic generalization as an alternative pathway to fear and avoidance [18], [27]. All groups had matched histories of directly experiencing shock following the CS+ and went on to show avoidance of, and give elevated threat belief ratings to, cues that acquired fear-provoking properties via verbal instructions or participation in derived equivalence relations. These results suggest that a direct history of both fear conditioning and avoidance learning is not necessary in order to show subsequent avoidance of potentially threatening stimuli. Interestingly, these findings indicate that fear conditioning and avoidance learning may be readily established on the basis of instructions alone. Further research that addresses these and other possibilities is warranted.

The present results are in line with the predictions of propositional models of avoidance learning [33], [34]. These models postulate that avoidance is driven by propositional beliefs about when aversive events are likely to occur and how they can be avoided. In accounting for the present findings, propositional models contend that during acquisition and testing the three pathways gave rise to similar propositions that resulted in equivalent levels of avoidance and modulation of threat beliefs. Provided that in all conditions, the same propositions are formed about the relations between CSs and the US and about the effect of avoidance behaviour on the US, behaviour should be identical in all conditions, irrespective of whether the propositions were based on experience, instructions, or inferences. The fact that avoidance responses were somewhat less frequent for the Derived CS+ than for the Instructed CS+ could be related to the fact that participants have to go through an additional inferential step in order to conclude that avoidance is necessary with a Derived CS+ (i.e., the proposition that the Derived CS+ is equivalent to a CS+ that is actually followed by the US). However, based on this reasoning, one would also have expected less avoidance for the Derived CS+ than for the Learned CS+, which was not observed. Hence, we refrain from making strong conclusions about whether or why the derived pathway leads to weaker avoidance than other pathways.

Our findings are also consistent with a modern functional account insofar as such an account also assumes only one underlying functional process common to all three learning pathways. More specifically, all of the research published to date on symbolically generalized fear and avoidance, assumes that a process known as the *transformation of stimulus functions* [37] underlies the generalization from CSs to those presented during critical test phases. This process is assumed to be a fundamental learning mechanism by which language comes to influence behaviour and is readily observed for verbally able populations, but not for non-verbal organisms [38]. Essentially, if at least two arbitrary stimuli, A and B, participate in a derived relation, such as equivalence, and A has a certain psychological function, like conditioned fear, then the functions of B will be transformed in accordance with that relation and also come to elicit fear, in the absence of further training. Of course, the findings of the Derived group are readily explained in these terms, but a similar theoretical analysis may be applied to the findings of the Instructed group as well. According to this view, verbal instructions such as “press the marked key to avoid shock when you see the blue circle” establish derived relations of equivalence between words (e.g., “blue circle”) and actual objects or events, while the words “press the marked key” alter the functions of the marked key such that the listener is likely to press in those instances or contexts specified in the rule (i.e., in the presence of the blue circle). To follow rules or instructions like this, the listener requires the ability to derive relations between words and objects and events, and it is in this way that the transformation of stimulus functions may be said to underlie the effects of instructions on avoidance behaviour. The transformation of functions can even be applied to explain directly conditioned fear or avoidance, insofar as the response functions of the US transfer to the CS (i.e., the stimulus properties of the CS are transformed by its contingent relationship to the US).

A functional, behaviour-analytic explanation of the current effects based on the transformation of stimulus functions offers the parsimony of a propositional account, whilst retaining its traditional emphasis on the prediction and control of behaviour without appeal to unobservable mental processes (i.e., it is atheoretical) [39]. It is important to note, however, that the functional and propositional accounts are not mutually exclusive [40]. Whereas the functional notion “transformation of stimulus functions” refers to the fact that stimulus functions can be transformed as the result of a particular learning history, propositional accounts focus on the mental processes by which stimulus functions can be altered (i.e., the formation and truth evaluation of propositions). Both types of accounts are thus directed at different levels of explanation and are therefore not mutually exclusive [40]. More generally, there is much to be gained from greater co-operation between functionally oriented and cognitively oriented researchers in explaining apparently unconditioned fear and avoidance, and given the clear overlap in research interests shared by these two traditions, such theoretical collaborations are long overdue [40].

The present study has some potential limitations. First, during extinction testing we could have presented a novel CS that was not involved in the learning phase and that was neither an instructed nor derived CS+. If participants always selected the avoidance response and expected the US after a novel CS, then it might suggest that effects observed were not due to instruction or derivation. Second, another variant of the procedures applied to the learned group might entail including a new CS in the avoidance learning phase and/or extinction test. Moreover, the derived group could also have been presented with a derived CS− to test whether non-avoidance is similarly shown to generalize like avoidance of a (derived) CS+. Third, future research should seek to examine whether the present findings are replicated when both the learned and derived groups are given filler training corresponding to the number of trials, rate of reinforcement and length of time taken by the derived group to complete the relational learning phase (e.g., with a yoked control procedure [41]). Finally, online shock expectancy ratings could have been measured on a trial-by-trial basis to chart the formation of propositional knowledge concerning the likelihood of shock modulated by the presence and absence of avoidance. Nevertheless, the present findings indicate, with a combination of within- and between-group comparisons, that the three pathways did result in clear and unambiguous levels of avoidance and threat-beliefs.

The present paradigm may be useful in the neuroimaging of avoidance. For instance, functional magnetic resonance imaging (fMRI) studies on the neural mechanisms of avoidance have focused exclusively on the directly learned pathway and identified a frontal-striatal-limbic network [42]–[44], with decreased activation in medial frontal and amygdala regions modulated by trait levels of experiential avoidance [45]. With regards to the present findings, it is likely that learned, instructed and derived pathways of threat-avoidance differentially recruit a frontal-striatal-limbic network. Although the expression of generalized fear involves the striatum, insula, thalamus/periacqueductal gray, and subgenual cingulate cortex [46], more research is needed on the neural mechanisms involved in the symbolic generalization of threat-avoidance and any potential overlap with fear generalization neurocircuity.
